# New Applications of Heparin and Other Glycosaminoglycans

**DOI:** 10.3390/molecules22050749

**Published:** 2017-05-06

**Authors:** Marcelo Lima, Timothy Rudd, Edwin Yates

**Affiliations:** 1 Department of Biochemistry, Federal University of São Paulo (UNIFESP), Vila Clementino, São Paulo, S.P. 04044-020, Brazil; 2 Department of Biochemistry, Institute of Integrative Biology, University of Liverpool, Crown Street, Liverpool L69 7ZB, UK; 3 National Institute of Biological Standards and Controls (NIBSC), Blanche Lane, Potters Bar, Herts EN6 3QG, UK

**Keywords:** heparin, glycosaminoglycans, chondroitin sulfate

## Abstract

Heparin, the widely used pharmaceutical anticoagulant, has been in clinical use for well over half a century. Its introduction reduced clotting risks substantially and subsequent developments, including the introduction of low-molecular-weight heparin, made possible many major surgical interventions that today make heparin an indispensable drug. There has been a recent burgeoning of interest in heparin and related glycosaminoglycan (GAG) polysaccharides, such as chondroitin sulfates, heparan sulfate, and hyaluronate, as potential agents in various applications. This ability arises mainly from the ability of GAGs to interact with, and alter the activity of, a wide range of proteins. Here, we review new developments (since 2010) in the application of heparin and related GAGs across diverse fields ranging from thrombosis and neurodegenerative disorders to microbiology and biotechnology.

## 1. Introduction

Heparin, a member of the glycosaminoglycan (GAG) family of sulfated polysaccharides, is one of the most widely used pharmaceuticals, whose major role is in the inhibition of clot formation and thrombi, especially during surgery or following trauma. Beyond referring to some important historical literature, this review will attempt to survey recent (by which we mean from ca. 2010 to 2017) applications for this important and widely used drug, and will include other GAG members, which are being explored increasingly for other potential uses.

Heparin has been an established anticoagulant drug for more than 60 years for the prevention and control of thrombotic events owing to its interaction with a number of proteins of the blood clotting cascade, notably antithrombin and thrombin [1]. It consists of a linear, highly sulfated polysaccharide chain of various lengths varying from 2000 to 40,000 Da [2,3,4], composed of repeating disaccharide units of 1,4 linked α-l-iduronic or β-d-glucuronic acid (d-GlcA), and α-d-glucosamine (d-GlcN). The predominant substitution pattern comprises 2-*O*-sulfation of the iduronate residues and *N*- and 6-*O*-sulfation of the glucosamine residues [5]. Heparin is also closely related structurally to heparan sulfate (HS), which is present on cell surfaces and in the extracellular matrix. HS is generally less sulfated than heparin and is often considered to have a defined domain structure [6,7,8] and a lower proportion of l-iduronate residues. There are also d-GlcA and N-acetylated glucosamine (d-GlcNAc) residues, as well as variations in sulfation, including a small proportion of 3-*O*-sulfates on glucosamine residues. Hyaluronic acid (HA) is the only member of the GAG family that is not sulfated, being a homo-polymer consisting of beta (1→4) linked disaccharides of d-GlcA β (1→3) d-GlcNAc. Chondroitin sulfate (CS) encompasses various structures, based on repeating β (1-4) linked disaccharides of GlcA β (1→3) GalNAc containing 6-(Chondroitin sulfate-C: CS-C) and 4-sulfates (CS-A), sulfation at position-2 of the GlcA residues (GlcA2S β (1→3) GalNAc6S) (CS-D), and 4,6-di-sulfated GalNAc (GlcA β (1→3) GalNAc4, 6diS (in CS-E). The polysaccharide, dermatan sulfate (DS), which was formerly known as CS-B, contains repeating →4) l-IdoA β (1→3) d-GalNAc β (1→ units with 4-*O*-sulfation on the GalNAc moiety. GAG-like structures from non-mammalian sources, often marine, have become the focus of attention recently, and these, while possessing similar backbone structures, often include branches of fucose units [9], which can include non-reducing terminal fucose with sulfation at positions-2 and -3 [10].

The discovery and development of heparin as an anticoagulant agent had a tremendous impact on health because major surgical procedures became possible, especially those in which cardiopulmonary bypass (CPB), which was first demonstrated in animals in 1939 [11], were necessary. Another important medical procedure that is only possible due to the anticoagulant properties of heparin is dialysis. Indeed, unfractionated (full-length) heparin (UFH) named heparin sodium, its antidote, protamine sulfate [12], as well as a form of low-molecular-weight heparin (LMWH) [13] all appear in the World Health Organization’s (WHO) List of Essential Medicines. The pharmacological activity of heparin results mainly from its ability to bind and accelerate the AT activity, thereby considerably enhancing the inhibition of coagulation factors Xa and IIa, although it interacts in concert with AT and other members of the blood clotting cascade as well, including factor IXa, XIa, and XIIa [14]. In a biological rather than a clinical context though, heparin action is more closely linked to defense against exogenous pathogens [15] and responses following tissue damage. Heparin alters cytokine levels [16], and one of its biological roles may be to dampen the effects of the sudden release of large numbers of cytokines following infection or sudden trauma.

In recent times, the majority of pharmaceutical heparin has been sourced from the intestine of pigs, and detailed studies of the sequence of heparin have involved laborious separation techniques. Recent advances in the biosynthesis of heparin-related structures employing recombinant enzymes and synthetic uridine-diphosphate-monosaccharide donors have been developed by Liu and Linhardt, allowing not only for the production of heparin sequences but also for the systematic and controlled preparation of heparin (and HS) analogues, which will be suitable for a wide-range of experimental purposes and applications [17].

## 2. Applications in Anticoagulation and Cancer Treatments

In current clinical use, one of the most important forms of heparin is low-molecular-weight heparin (LMWH) which consists of a complex mixture of fragments ranging from tetra to hexadecasaccharides and somewhat higher oligosaccharides [18,19,20] obtained by various chemical and enzymatic depolymerization processes. The main advantages of LMWHs over UFH are improved bioavailability and higher anti-factor Xa/anti-factor IIa activity ratios, with decreased hemorrhagic risk during prolonged treatments [21]. While UFH can be monitored effectively and reversed in patients undergoing surgery with extracorporeal circulation, LMWHs cannot, because neutralization with protamine sulfate is ineffective. The development of new anticoagulant agents with the beneficial properties of both UFH and LMWH is therefore still pursued and is an important area in which biosynthetic structures are being applied [22,23].

Aside from the well-known anticoagulant activities of heparin and related GAGs or GAG mixtures, such as sulodexide (a mixture of LMWH and DS in the ratio 80:20), heparin is also active in a wide range of activities in which the naturally occurring cell surface polysaccharide, heparan sulfate (HS), is a participant. Many of the proteins with which heparin interacts originate in the extracellular matrix, the best studied of which belong to the fibroblast growth factor (FGF) family. The potential use of heparin to interfere with aberrant cell–cell signaling by this route has been investigated and the relationship between sequence and activity sought [24,25,26,27].

The importance of the FGF signaling system to development and regulation, and hence when its function is impaired or altered, also to disease is one obvious area in which heparins, acting in this case as analogues for HS, can be applied. One area in which modulating FGF signaling would be desirable is cancer treatment. However, it is noteworthy that cancer-related thrombotic disorders are also well known and were first described as long ago as 1865, when the clinical association between thrombosis and an initially undiagnosed cancer was noted. Unfractionated heparin and LMWHs have been used successfully for the prophylaxis and treatment of cancer-related hemostatic disorders; however, since cancer patients have an increased risk of bleeding [28], LMWHs have gained significant ground since they produce a more predictable anticoagulant response, display improved subcutaneous bioavailability, and exhibit an extended half-life. Furthermore, heparin-induced thrombocytopenia (HIT) incidence is lower when LMWHs and the synthetic pentasaccharide, Fondaparinux, are used. Novel oral anticoagulants are under development, but their efficacy and safety in cancer-patients has yet to be proved. Despite being well recognized now, the pathogenesis is complex and results from the combination of several factors, but the expression of tumor cell-associated clotting factors is a shared characteristic. The prevention and treatment of these conditions extends beyond the ease of symptoms, it has a direct impact in cancer patient survival [29], and there are some known beneficial effects of heparin, but elucidating these is hampered by the complexity of the systems involved. The widespread use of heparin as a treatment is hindered by unwanted anticoagulant side-effects [30] (see also review by Afratis et al. [31]); however, the possibility of correcting signaling defects caused by altered HS structure (e.g., by sulf enzyme activity) in cancer [32,33] or the direct inhibition of the sulf enzymes with heparin or heparin analogues is an area of potential interest. Of direct relevance to the progression of the disease is the inhibition of a mammalian heparanase enzyme [34], while other routes are thought to influence metastasis, acting via P- and L-selections [35,36,37], inhibiting galectins [38], inhibiting tumorogenesis and angiogenesis through cellular receptors such as CD44 and growth factor receptors [39], or, in the case of the use of heparin as a means of ameliorating resistance to cisplatin treatment, acting through as-yet unidentified mechanisms [40], all of which are affected by heparin or heparin analogues. Among other GAGs, an example of the treatment of cancer is the use of the non-sulfated GAG, hyaluronate (HA) in pancreatic ductal adenocarcinoma [41].

## 3. Recovery from Nervous System Damage

Recent work shows that, while CS inhibits the regeneration of neurites in the CNS, CS-bound HB-GAM (pleiotrophin) activates them, inducing dendrite regeneration in adult cerebral cortex and axonal regeneration in adult spinal cord [42]. Chondroitin sulfate forms a barrier following nerve injury [43], but can be digested by enzymes to improve repair [44,45,46,47]. Chondroitin sulfate-E (CS-E), containing unusual 4,6 di-sulfated GalNAc residues and 4-sulfated CS in aggrecan, have been shown to be inhibitory to neurite outgrowth [48], and CS-E mediates estrogen-induced osteoanabolism [49], suggesting several potential future applications for CS and its derivatives.

## 4. Respiratory Diseases

GAGs and heparin in particular have been suggested as playing a protective roles in the inflammation response [50], which has been interpreted as involving (among other things) the inhibition of elastase and the interaction with several cytokines [51]. Heparin and its analogues have been proposed for the application to elastase inhibition for cystic fibrosis treatment (or other conditions, such as acute respiratory distress syndrome (ARDS). Although they do not originate from a conventional mammalian GAG, oligosaccharide fragments of the fucosylated GAG structures from the sea cucumber (*Holothuria forskali*) [9] were shown to lower neutrophil infiltration by reducing selectin interactions.

## 5. Neurodegenerative Diseases

There is an emerging role in Parkinson's Disease for heparin acting via cathepsin-d activity affecting α-synuclein accumulation [52], while, in Alzheimer’s Disease, the inhibition of BACE-1, the key protease responsible for the generation of toxic Aβ fragments (1–42) is inhibited by heparin and its derivatives [53,54], some of which have been engineered to possess very low anticoagulant activity [55,56]. Furthermore, it is known that heparin modulates fibril formation [57], and understanding the mechanisms underlying such events may open new routes for the prevention, for instance, of toxic fibril formation and deposition.

## 6. Roles as Antimicrobial Agents

### 6.1. Viruses

GAGs might be expected, on the basis of their universal presence on cell surfaces, to serve as a broad spectrum and relatively non-specific receptors for virus binding [58]; [see also an earlier review: [59]]. Such interactions are not unexpected, since virus envelope proteins present patches of positively charged amino acids. While GAGs have been shown to attach to filoviruses [60] and CS-E, but not CS-D, and inhibit dengue virus infection [61], it is clear that GAGs are indeed of relevance to the mechanisms of viral attachment and invasion. The ability of viruses to bind cell surface HSs and, by extension, bind heparin under experimental conditions has long been known in the case of herpes simplex (HSV) and dengue (DENV) viruses. Heparin as a potential inhibitor of viral attachment follows naturally from these observations and has been demonstrated for DENV [62]. What is less clear is whether and how this knowledge can be exploited to enable GAGs to be applied as inhibitors of attachment, a process that may be able to be exploited for the well-known property of multivalency to increase the avidity of the binding of the viral particles to the GAG, perhaps immobilized in some device, or attached to multidentate ligands in a nanotechnology format (see also a review of this field: [63]). The potential importance of such applications is difficult to over-emphasize, however. Recently, viral attachment to GAGs was reported for IFNα/βBP from variola (smallpox virus), monkeypox viruses [64], and GAGs have been shown to prevent measles virus infection in cell lines via hemagglutinin protein [65]. Other viruses, including hepatitis B, have been found to bind to heparin [66] as has Japanese encephalitis virus (JEV) [67]. Thus, the interest in GAG-based intervention seems set to increase.

Heparin and derivatives have been shown to be effective in preventing the infection of cells by the influenza virus, strain H5N1 [68], and to have effects on ZIKV-induced cell death that are independent of adhesion and invasion [69]. In this case, heparin has only a modest ability to protect infection by analogy with dengue virus, which is also of the flavivirus family, and which interacts with heparin through the envelope glycoproteins [70]. Rather, heparin may be acting to protect infected cells from cytotoxic effects and cell death via the activation of cell survival signaling pathways. The ability of heparin to protect cells from programmed cell death had already been observed in human cells (non-infected) [71], and this may form an interesting new avenue for the application of GAG derivatives in itself.

### 6.2. Parasites

The involvement of GAGs in a parasitic disease has been studied relatively little. However, the potential for the application of GAGs remains high in cases where GAGs form a major part of the means of attachment and invasion, especially where there is the possibility of topical application. The ability of heparin and derivatives to inhibit rosetting of parasite infected erythrocytes has been noted [72] and, recently, effective inhibitors (comprising fucosylated CS (FucCS)) of cytoadherence (the process of adhesion of infected red blood cells to vascular epithelia) derived from sea cucumber have been reported [73]. Heparin is known to alter the activity of a cysteine protease in the parasite, *L. mexicana,* that causes Leishmaniasis [74] and interacts with a metalloprotease from *L.(v). braziliensis* [75]. Heparin has also been shown to accelerate protein degradation by human neutrophil elastase [76]. Several heparin-binding proteins have also been identified in *T. cruzi,* where some of them are implicated in parasite adhesion to midgut epithelial cells [77].

### 6.3. Bacteria

Binding of GAGs facilitates Streptococcal entry into the CNS of *Drosophila* [78] and determines disease progression [79]. In relation to the phenomenon of shock, GAGs seem to be elevated overall, perhaps originating from damaged tissue, but they also seem to neutralize antimicrobial peptides [80]. Glycosaminoglycans were studied en masse, however, without paying attention to the individual types involved. Heparin use in sepsis patients, where crosstalk between thrombosis and inflammation plays a critical role, has also been recognized. The use of heparin prevents the development of microthromboembolic disease during sepsis, impairing tissue hypoxygenation and further organ damage and dysfunction [81]. There is also literature pointing to the use of heparin-coated stents for the prevention of bacterial biofilm formation and subsequent microbe attachment [82].

## 7. Panceatitis

Acute pancreatitis has been reported independently by several groups to benefit from treatment with heparin [83] in rats and in patients [84,85,86]. Furthermore, LMW heparin has also been shown to be effective [87].

## 8. Roles in Rheumatoid Arthritis (RA)

Naturally occurring autoantibodies to GAGs are markers in rheumatoid arthritis [88]. The level of IgM type Ab for CS-C in serum correlates with RA, although GAG concentration alone is not a good indicator in knee injury [89] in agreement with earlier findings in horses [90].

## 9. Inflammation Reduction

Many of the applications of heparin mentioned in this review may stem, at least in part, from the ability of heparin to moderate inflammation. This topic has itself been reviewed in 2013 [91] and, in general, heparin is perceived as acting via several routes, which include interaction with cytokines and as an inhibitor of heparanase, to achieve reduced leukocyte recruitment.

Applications in which these properties may be important include the treatment of burns [92] and the proposed use of heparin in asthma treatment [93]. The potential, based on a projection of the ability of heparin to inhibit bacterial infection and reduce inflammation, to improve the symptoms of cystic fibrosis has also been investigated [94] but no marked improvement at low doses (50,000 IU) has been shown.

While cystic fibrosis applications acting to reduce inflammation assist sputum clearance, inhibit elastase, or act as an inhibitor of microbial attachment or growth have not been convincing, heparin derivatives were shown to be capable of acting at several points in the inflammatory process [51].

## 10. Alternative Sources of GAG-Like Structures with Potentially Useful Activities

This review attempts to cover not only new applications for heparin but also potential new sources of active agents among the GAGs for both old and new applications. Although not all of these structures correspond to GAGs according to the classical definitions of mammalian GAGs provided above, GAG-like structures are found in a wide range of organisms, including *Cnidaria*, *Arthropoda*, *Mollusca*, *Echinodermata*, and *Chordata* [95]. A host of polysaccharides, either GAGs in the conventional sense or polysaccharides with structural features closely resembling GAGs, usually bearing additional ramifications have been identified and linked to a variety of biological activities. These include Sea cucumber (*S. hermanni*) as an antiinflammatory [95], marine GAG analogues [96], fish cartilage as an antitumor and anti-pathognic agent [97], GAGs from starfish with antithrombotic and anti-inflammatory activities [95], and mussels (*Perna canaliculis*) as an anti-inflammatory agent [98].

## 11. Biotechnological and Other Applications

An emerging use of GAGs is in biotechnological applications, particularly in the field of embryonic stem cells, for the regulation of differentiation [reviewed in [99]] and neural speciation in mouse embryonic stem cells [100]. The successful fractionation of a cell population by HS epitopes has also been reported [101]. The prospect of incorporating GAGs into electrospun meshes for the purpose of supporting stem cell applications has also been undertaken [102]. A possible role is emerging in muscular dystrophy, following signs of involvement of HS sulfation in the disease process and ageing, acting via FGF-2 [103].

## 12. Concluding Remarks

Many a student textbook of biochemistry once dismissed the role of GAGs as merely structural; decades of intensive research led to a burgeoning of the biological roles ascribed to them and potential practical applications. The precise nature of the relationship between their structure and function remains a matter of debate, a fact that stems not only from their structural complexity, but also the difficulty of analysis, which may also indicate that new approaches are required. These approaches may need to be sensitive to as yet undefined properties, and one such property is their large-scale vibrational modes [104]. Nevertheless, even without this detailed information, it is clear that the number of roles of these versatile materials seem to set to increase in the future.
